# Type-I band alignment of BX–ZnO (X = As, P) van der Waals heterostructures as high-efficiency water splitting photocatalysts: a first-principles study

**DOI:** 10.1039/d0ra09701b

**Published:** 2020-12-17

**Authors:** Thi-Nga Do, M. Idrees, Nguyen T. T. Binh, Huynh V. Phuc, Nguyen N. Hieu, Le T. Hoa, Bin Amin, Hieu Van

**Affiliations:** Laboratory of Magnetism and Magnetic Materials, Advanced Institute of Materials Science, Ton Duc Thang University Ho Chi Minh City Vietnam dothinga@tdtu.edu.vn; Faculty of Applied Sciences, Ton Duc Thang University Ho Chi Minh City Vietnam; Department of Physics, Hazara University Mansehra 21300 Pakistan; Department of Fundamental Sciences, Quang Binh University Quang Binh Vietnam; Division of Theoretical Physics, Dong Thap University Cao Lanh 870000 Vietnam; Institute of Research and Development, Duy Tan University Da Nang 550000 Vietnam lethihoa8@duytan.edu.vn; Faculty of Natural Sciences, Duy Tan University Da Nang 550000 Vietnam; Abbottabad University of Science and Technology Abbottabad 22010 Pakistan; Department of Physics, University of Education, The University of Da Nang Da Nang Vietnam

## Abstract

In this work, we perform first-principles calculations to examine the electronic, optical and photocatalytic properties of the BX–ZnO (X = As, P) heterostructures. The interlayer distance and binding energy of the most energetically favorable stacking configuration are 3.31 Å and −0.30 eV for the BAs–ZnO heterostructure and 3.30 Å and −0.25 eV for the BP–ZnO heterostructure. All the stacking patterns of the BX–ZnO heterostructures are proved to have thermal stability by performing AIMD simulations. The BAs–ZnO and BP–ZnO heterostructures are semiconductors with direct band gaps of 1.43 eV and 2.35 eV, respectively, and they exhibit type-I band alignment, which make them suitable for light emission applications with the ultra-fast recombination between electrons and holes. Both the BAs–ZnO and BP–ZnO heterostructures can exhibit a wider optical absorption range for visible-light owing to their reduced band gaps compared with the isolated BAs, BP and ZnO monolayers. The band alignment of both the BAs–ZnO and BP–ZnO heterostructures can straddle the water redox potential and they would have better performances owing to the direct band gap and the reduced band gap. All these findings demonstrate that the BX–ZnO heterostructures can be considered as potential photocatalysts for water splitting.

## Abstract 2

In this work, we perform first-principles calculations to examine the electronic, optical and photocatalytic properties of the BX–ZnO (X = As, P) heterostructures.

## Graphical abstract

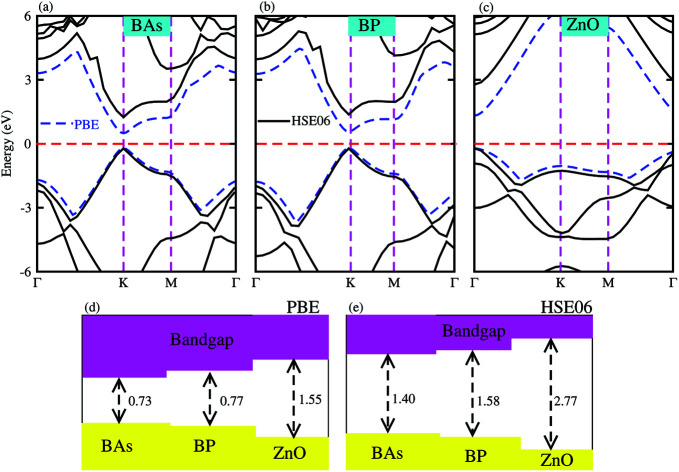

## I. Introduction

Recently, the combination of two or more two-dimensional materials (2DMs) to establish van der Waals (vdW) heterostructures has been proved to be one of the most common strategies to enhance the electronic, optical and photocatalytic properties of 2DMs, which will affect mainly the performances of 2DMs-based nanodevices.^1–4^ Thus, the formation of vdW heterostructures can improve the devices’ performances and extend the range of applications of 2DMs. In this way, it is interesting that many prospective properties and new findings that do not exist in single 2DMs can appear from the vdW heterostructures. Nowadays, there have been several vdW heterostructures that have been fabricated in experiments and proposed theoretically.^5–11^ Generally, vdW heterostructures can be obtained easily in experiments by various methods, including mechanical assembly and direct growth.^12–14^ Whereas, the vdW heterostructures can be predicted theoretically by stacking 2DMs on top of others.^15–17^ It is clear that the 2DMs are held together in their vdW heterostructures by the weak vdW interactions, which keep the vdW heterostructures energetically stable and give rise to easy exfoliation. All the above findings demonstrate that the vdW heterostructures have emerged as promising candidates for a variety of high-performance optoelectronic and nanoelectronic devices.^18–21^

In recent years, the zinc oxide (ZnO) monolayer which was successfully synthesized in experiments^22,23^ has attracted more interest from the scientific community. ZnO exhibits direct band gap semiconductor behaviour.^24^ The electronic properties of the ZnO monolayer can be enhanced by several strategies, including strain,^25^ functionalization,^26^ electric field.^27,28^ The controllable electronic properties of this material make it a promising candidate for field-effect transistors and photovoltaic devices.^29,30^ Boron arsenide (BAs), a new type of group III–V semiconductor, has recently also attracted considerable interest owing to its ultra-thin thermal conductivity.^31^ The electronic and thermal features of bulk and 2D BAs have been also investigated theoretically.^32–34^

The vdW heterostructures based on the ZnO monolayer as well as BX (X = As, P) have recently gained special attention from researchers and they have been predicted to be promising candidates for designing excellent nanoelectronic and optoelectronic devices. For instance, Gao and colleagues^35^ predicted that the ZnO–GeC heterostructure can be considered as having potential for photocatalytic devices owing to its enhanced optical absorption and the formation of the type-II band alignment. Liu *et al.*^36^ proposed the BP/AsP heterostructure within first-principles calculations and demonstrated that this heterostructure is expected to have great potential applications in photovoltaic devices and photocatalysis. Wang and colleagues^6^ showed that the ZnO/MoS_2_ heterostructure exhibits a strong optical absorption, which makes this heterostructure have potential for photocatalytic applications. The type-I band alignment in the BAs/MoS_2_ heterostructure makes it suitable for photocatalysis.^37^ More interestingly, to our best knowledge, the combination between BX (X = As, P) and ZnO monolayers has not yet been proposed and investigated. This combination promises several interesting properties that may not exist in the parent monolayers and may satisfy for future excellent optoelectronic and nanoelectronic applications. Therefore, in this work, we perform first-principles calculations to examine the electronic, optical and photocatalytic properties of the BX–ZnO heterostructures. Our results could provide basic guidance for practical applications of such heterostructures for future nanoelectronic and optoelectronic devices.

## II. Computational methods

In this work, all the atomic optimization and electronic characteristics calculations are performed by a first-principles calculation within density functional theory (DFT). To perform all these calculations, we used the simulated Quantum Espresso.^38^ The Troullier–Martins norm-conserving pseudopotential is employed to understand the valence state of electrons. The exchange correlation energy is approximated by the generalized-gradient approximation within the Perdew–Burke–Ernzerhof (PBE) pseudopotential.^39^ The PBE method predicts correctly the physical trends of materials, but it underestimates the band gap. Therefore, the Heyd–Scuseria–Ernzerhof hybrid functional^40,41^ was introduced to obtain a more accurate band gap and optical properties of the materials. All calculations are performed after the geometry was optimized until all residual forces on each atom are smaller than 0.01 eV Å^−1^. To eliminate the boundary interactions along the *z* direction, we applied a large vacuum thickness of 40 Å. To correctly describe the weak forces in the layered materials, we used the corrected dispersion approximation DFT-D3.^42^

## III. Results and discussion

**Fig. 1:**
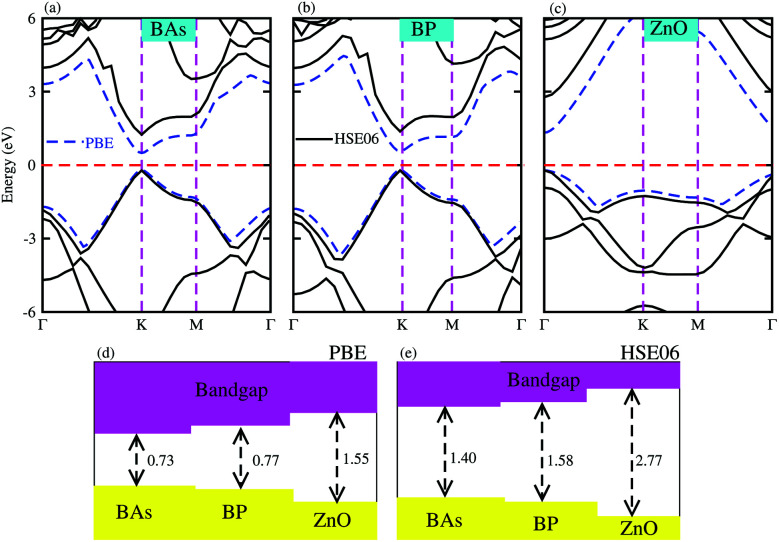
Band structures of pristine (a) BAs, (b) BP and (c) ZnO monolayers calculated by PBE and HSE06 methods. The solid black lines and the dashed blue lines represent the HSE06 and PBE band structures, respectively. The dashed red lines stand for the Fermi energy level, which is set to be zero. The calculated band gaps of monolayers obtained from (d) PBE and (e) HSE06 methods.

Firstly, we calculated the structural and electronic properties of pristine BAs, BP and ZnO monolayers, as depicted in Fig. 1. Our results show that the lattice constants of pristine BAs, BP and ZnO monolayers are calculated to be 3.39 Å, 3.21 Å and 3.29 Å, respectively. The calculated band gaps of pristine BAs, BP and ZnO monolayers are 0.73/1.40, 0.77/1.58 and 1.55/2.77 eV, respectively, obtained from the PBE/HSE06 method, as illustrated in Fig. 1(d) and (e). One can find that both the PBE and HSE06 methods predict the same nature in the band structures of these monolayers. The main difference in the PBE and HSE06 band structures is the shifting of the conduction band minimum (CBM) towards the higher binding energy. Both the PBE and HSE06 methods predict direct band gap semiconductors of BAs, BP and ZnO monolayers, as shown in Fig. 1(a)–(c). All these findings are in good agreement with previous reports.^32,35^ This demonstrates that our computational models and methods are reliable and accurate.

**Fig. 2:**
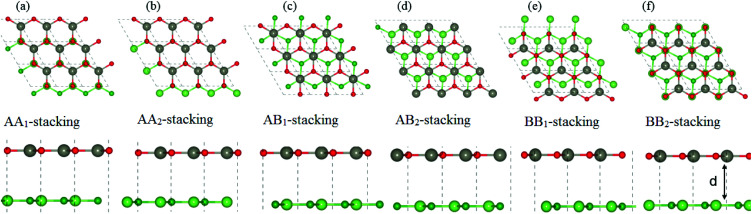
Top view (top panel) and side view (bottom panel) of the atomic structures of BX–ZnO heterostructures for six different possible stacking configurations. Red and gray balls represent the O and Zn atoms, whereas green and dark green represent the X and B atoms, respectively.

Next, we construct the heterostructures, composed of 2D BX (X = As, P) and ZnO monolayers. As the lattice constants of BAs, BP and ZnO monolayers show very small lattice mismatch (BAs–ZnO shows 2.9% and BP–ZnO shows 2.4%), they are suitable to design a vdW heterostructure, having a precise stacking configuration. Therefore, for making BAs–ZnO and BP–ZnO vdW heterostructures, we consider six possible stacking patterns for each vdW heterostructure, as presented in Fig. 2. First, the calculated interlayer distances between BX and ZnO in their BX–ZnO heterostructures for the different stacking patterns are ranging from 3.31 Å to 3.49 Å. These values of the interlayer distances are comparable to those of other typical van der Waals crystals, such as graphite,^43^ BlueP–GeC,^44^ GaN–BSe^45^ and so forth. Second, one can find that the covalent radii of B, As (P), Zn, and O are shown to be 0.82, 1.19 (1.06), 1.25 and 0.73 Å, respectively. The sum of the covalent radii of B and O and Zn and As (P) are 1.55 and 2.44 (2.31) Å, respectively, which are still smaller than the calculated interlayer distances of the considered heterostructures, implying that there is no chemical bonding between the BX and ZnO layers. Based on these, we can conclude that the BX and ZnO layers are bonded together *via* the weak vdW interactions. To check the stability of all stacking patterns, we further calculate their binding energies as follows:

1 *E*_b_ = *E*_BX–ZnO_ − *E*_BX_ − *E*_ZnO_

**Fig. 3:**
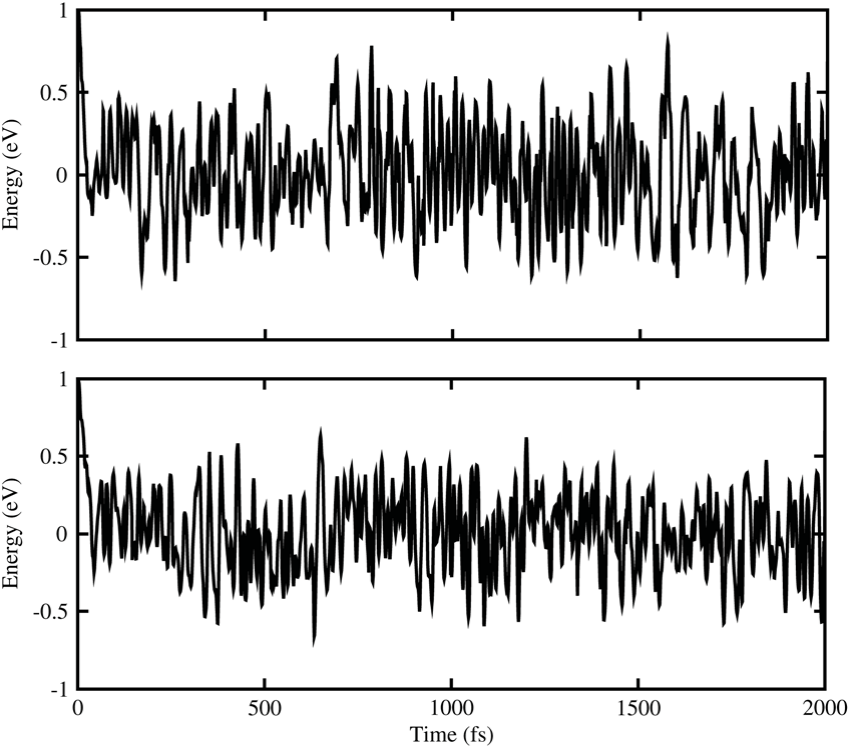
The energy fluctuations of (top) BAs–ZnO and (bottom) BP–ZnO heterostructures as a function of time step by performing the AIMD simulation within 2 ps at room temperature (300 K).

Here, *E*_BX–ZnO_, *E*_BX_ and *E*_ZnO_ are the total energies of the BX–ZnO heterostructure, isolated BX and ZnO monolayers, respectively. The interlayer distances and binding energies of the BX–ZnO heterostructures for all stacking patterns are listed in Table 1. One can find from Table 1 that the AB_1_-stacking pattern has the smallest interlayer distance and the lowest binding energy compared with other stacking patterns. It is interesting that the lower the binding energy is, the more stable the heterostructure can be established. This finding indicates that the stacking pattern (c) is the most stable one. Furthermore, *ab initio* molecular dynamics (AIMD) simulations for BAs–ZnO and BP–ZnO vdW heterostructures are also performed to examine their thermal stability, as illustrated in Fig. 3. It is clear that there are small impacts on the energy of the BAs–ZnO and BP–ZnO vdW heterostructures before and after heating up to 2000 fs. This suggests that both the BAs–ZnO and BP–ZnO heterostructures are thermally stable at room temperature (300 K).

**Table 1:** Calculated interlayer distances and binding energies of the BX–ZnO heterostructures for six different stacking patterns

	*E*_b_, eV		*d*, Å	
	BAs–ZnO	BP–ZnO	BAs–ZnO	BP–ZnO
AA_1_-stacking	−0.23	−0.19	3.42	3.35
AA_2_-stacking	−0.27	−0.13	3.39	3.38
AB_1_-stacking	−0.30	−0.25	3.31	3.30
AB_2_-stacking	−0.21	−0.15	3.45	3.36
BB_1_-stacking	−0.19	−0.17	3.49	3.32
BB_2_-stacking	−0.28	−0.11	3.35	3.40

**Fig. 4:**
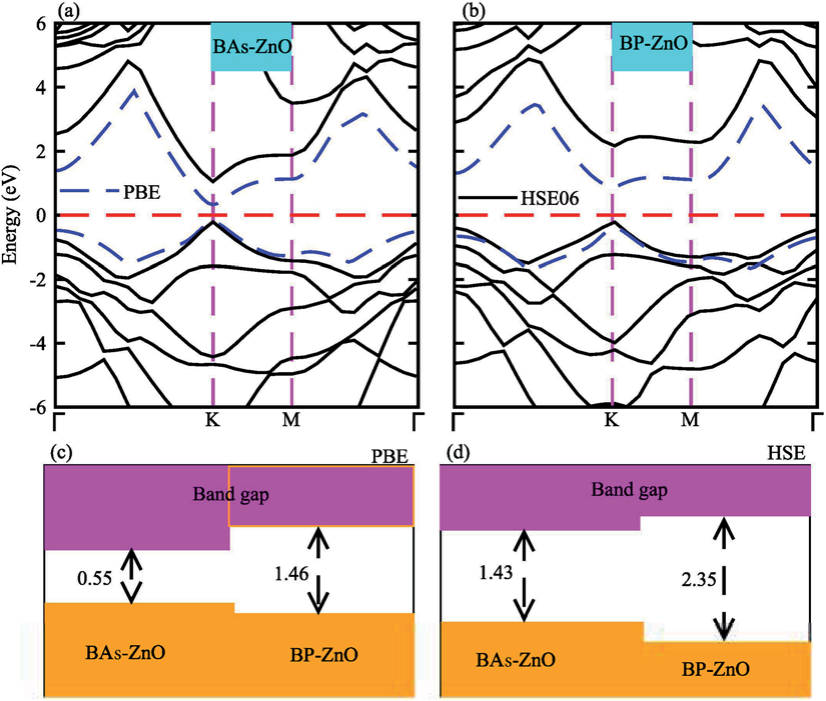
Calculated band structures of (a) BAs–ZnO and (b) BP–ZnO heterostructures by PBE and HSE06 calculations. The band gaps of BAs–ZnO and BP–ZnO heterostructures are calculated by (c) PBE and (d) HSE06 methods.

The electronic band structures and band gaps of the BAs–ZnO and BP–ZnO vdW heterostructures are displayed in Fig. 4 using HSE06 and PBE calculations. One can find that the BAs–ZnO vdW heterostructure is a direct band gap semiconductor. Both the VBM and CBM of this heterostructure are located directly at the *K* point, as depicted in Fig. 4(a). The calculated band gap of the BAs–ZnO vdW heterostructure is 0.55/1.43 eV as obtained from the PBE/HSE06 method. This band gap is rather smaller than that of the pristine BAs and ZnO monolayers. This demonstrates that constructing the BAs–ZnO vdW heterostructure gives rise to a decrease in the band gap of semiconductors. Similar to the BAs–ZnO heterostructure, the BP–ZnO heterostructure also is a direct band gap semiconductor with both the CBM and VBM at the *K* point. The band gap of such a heterostructure is calculated to be 1.46/2.35 eV using PBE/HSE06 calculations. This band gap of the BP–ZnO heterostructure is larger than that of the BP monolayer but smaller than that of the ZnO monolayer using both the PBE and HSE06 calculations. The direct band gap nature in both the BAs–ZnO and BP–ZnO heterostructures makes them promising materials for the fabrication of optoelectronic devices. Furthermore, we can find that the band gaps of both the BAs–ZnO and BP–ZnO heterostructures are still larger than the band gap requirement for photocatalysis reactions, implying the potential application as a visible light photocatalyst.

**Fig. 5:**
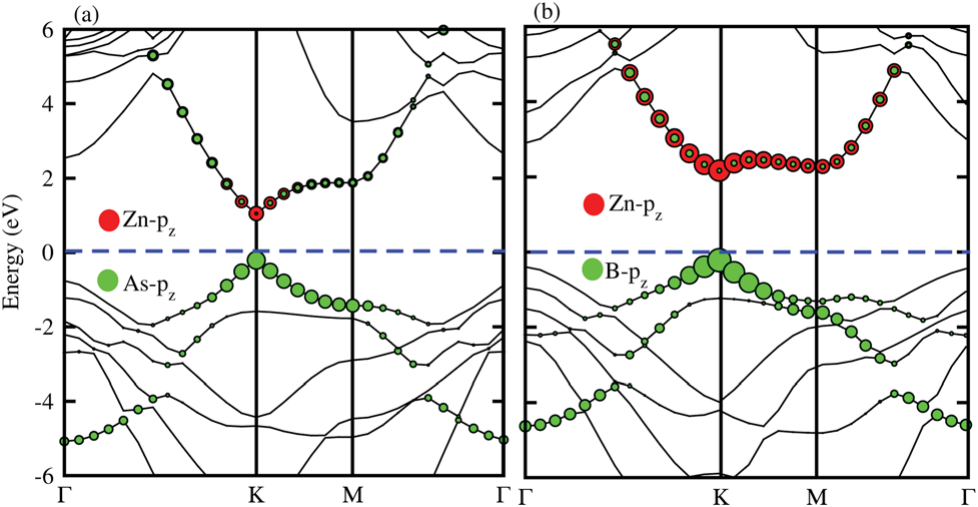
Weighted band structures of (a) BAs–ZnO and (b) BP–ZnO vdW heterostructures using HSE06 calculations.

In order to determine the formation of the band alignment (type-I, type-II or type-III) in the BX–ZnO heterostructures, we further calculate the weighted band structures of the BAs–ZnO and BP–ZnO vdW heterostructures. These results are depicted in Fig. 5(a) and (b) for the BAs–ZnO and BP–ZnO heterostructures, respectively. From the weighted band structure of the BAs–ZnO heterostructure in Fig. 5(a), we find that both the VBM and CBM of such a heterostructure is due to the As-p_*z*_ state of the BAs layer. It demonstrates that the BAs–ZnO heterostructure forms the type-I band alignment. Furthermore, all these orbital overlaps can modify the orbitals and enhance the optical performance. Similar to the BAs–ZnO heterostructure, the BP–ZnO heterostructure also possesses type-I band alignment. This formation can be found in Fig. 5(b).

**Fig. 6:**
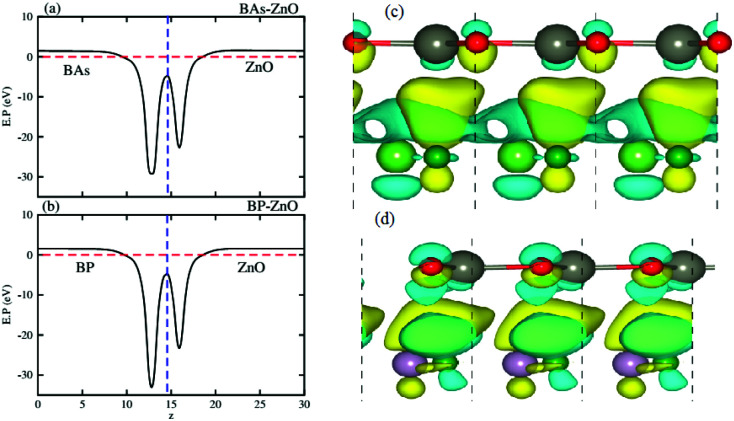
Electrostatic potentials of (a) BAs–ZnO and (b) BP–ZnO heterostructures along the *z* direction. The charge density differences of (c) BAs–ZnO and (d) BP–ZnO heterostructures. The yellow and cyan regions represent the charge accumulation and depletion, respectively.

Fig. 6(a) and (b) represent the electrostatic potentials of the BX and ZnO layers in their corresponding heterostructures along the *z* direction. It is evident that both the BAs and BP layers exhibit a deeper potential than the ZnO layer in their corresponding heterostructure. This gives rise to the formation of a built-in electric field, pointing from the BX layer to the ZnO layer. It is interesting that owing to the formation of such a built-in electric field, the shifts in carrier can be promoted and it leads to the reduction of the recombination of photogenerated electron–hole pairs. To further understand the charge redistribution in the BX–ZnO heterostructures, we calculate the charge density difference between the BX layers and the ZnO layer as follows:

2 Δ*ρ* = *ρ*_BX–ZnO_ − *ρ*_BX_ − *ρ*_ZnO_

**Fig. 7:**
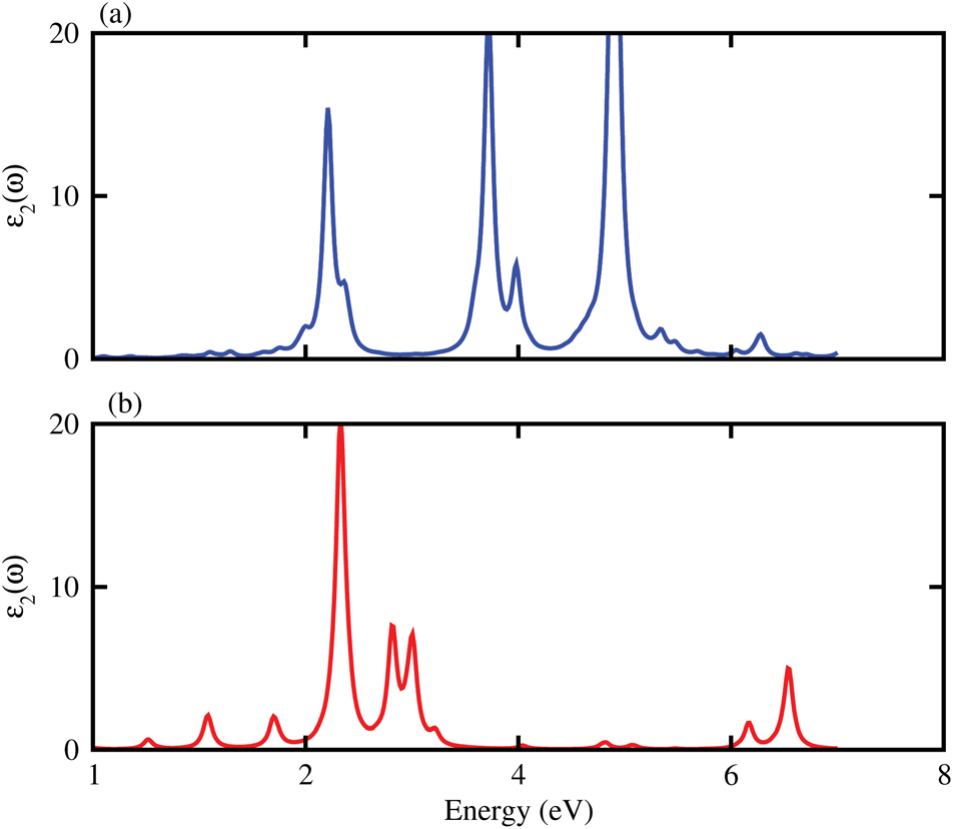
Calculated imaginary part of dielectric functions of BAs–ZnO and BP–ZnO heterostructures.

Here, *ρ*_BX–ZnO_, *ρ*_BX_ and *ρ*_ZnO_ represent the charge densities of the BX–ZnO heterostructure, isolated BX and ZnO layers, respectively. The charge density differences of the BAs–ZnO and BP–ZnO heterostructures are displayed in Fig. 6(c) and (d), respectively. The yellow region represents the positive value of charges, whereas the cyan region stands for the negative value. The charge redistribution is mainly visualized at the interfaces of heterostructures. By Bader charge analysis, we find that there is only 0.012*e* transferred from the ZnO to BAs layer, while about 0.029 electrons are transferred from the ZnO to BP layers. We further investigate the optical properties of the BAs–ZnO and BP–ZnO heterostructures by calculating the imaginary part (*ε*_2_(*ω*)) as a function of photon energy, as shown in Fig. 7. One can expect that both the BAs–ZnO and BP–ZnO heterostructures can exhibit a wider optical absorption range for the visible-light owing to their reduced band gaps compared with the isolated BAs, BP and ZnO monolayers.

The photocatalytic response of BAs, BP, ZnO, and their corresponding BAs–ZnO and BP–ZnO vdW heterostructures for water splitting at pH = 0 is calculated using Mulliken electronegativity (*χ*) as follows:

3 *E*_VBM_ = *χ* − *E*_elec_ + 0.5*E*_g_

and,

4 *E*_CBM_ = *E*_VBM_ − *E*_g_

**Fig. 8:**
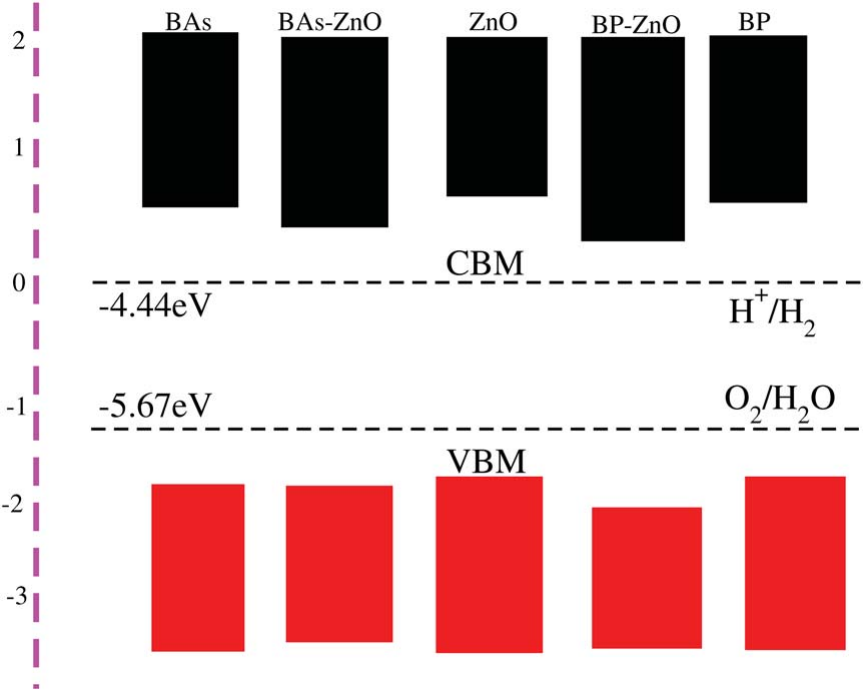
The band alignments of the BAs, BP, ZnO monolayers and their corresponding BAs–ZnO and BP–ZnO heterostructures using HSE06 calculations with respect to the standard oxidation (−5.67 eV) and reduction (−4.44 eV) potentials for water splitting.

Here, *E*_g_ represents the band gap of materials. *E*_elec_ is the energy of free electrons in the hydrogen scale (4.5 eV). The band alignments of BAs, BP, ZnO and the corresponding BAs–ZnO and BP–ZnO heterostructures are depicted in Fig. 8. The reduction and oxidation potentials are obtained from the value of the pH as: *E*_reduction_ = −4.44 eV + pH × 0.0059 eV, and *E*_oxidation_ = −5.67 eV + pH × 0.0059 eV. It should be noted that there are two main requirements for a material that is considered as a promising candidate for an efficient photocatalyst. First, this material should have a band gap from 1.23 eV to 3.0 eV. Second, the band edge positions of this material must straddle the water redox potentials. It requires that the potential of the CBM of materials should be higher than −4.44 eV and the potential of the VBM should be lower than −5.67 eV. The band gaps of the BAs–ZnO and BP–ZnO heterostructures are calculated to be 1.43 eV and 2.35 eV, respectively. These values of the band gaps of the heterostructures are in the range from 1.23 to 3.0 eV, thus they meet the first requirement for an efficient photocatalyst. One can find from Fig. 8 that the band alignment of both the BAs–ZnO and BP–ZnO heterostructures can straddle the water redox potential. One more interesting point, which should be highlighted here, is that compared with the BAs, BP and ZnO monolayers, both the BAs–ZnO and BP–ZnO heterostructures would have better performances owing to the direct band gap and the reduced band gap.

## IV. Conclusions

In conclusion, we have investigated the electronic, optical and photocatalytic properties of the BX–ZnO (X = As, P) heterostructures by performing first-principles calculations. Our results demonstrate the interlayer distance and binding energy of the most energetically favorable stacking configuration are 3.31 Å and −0.30 eV for the BAs–ZnO heterostructure and 3.30 Å and −0.25 eV for the BP–ZnO heterostructure. All the stacking patterns of BX–ZnO heterostructures are proved to have thermal stability by performing AIMD simulations. Both the BAs–ZnO and BP–ZnO heterostructures are semiconductors with direct band gaps of 1.43 eV and 2.35 eV, respectively, by HSE06 calculations. Both the VBM and CBM of the BX–ZnO heterostructures come from the As-p_*z*_ or B-p_*z*_, confirming the formation of the type-I band alignment. Both the BAs–ZnO and BP–ZnO heterostructures can exhibit a wider optical absorption range for visible-light owing to their reduced band gaps compared with the isolated BAs, BP and ZnO monolayers. The band alignment of both the BAs–ZnO and BP–ZnO heterostructures can straddle the water redox potential and they would have better performances owing to the direct band gap and the reduced band gap. All these findings demonstrate that the BX–ZnO heterostructures can be considered as potential photocatalysts for water splitting and provide theoretical guidance for designing high-performance nanoelectronic and optoelectronic devices based on the BX–ZnO heterostructures.

## Conflicts of interest

There are no conflicts to declare.

## Supplementary Material
